# The impact of COVID-19 social disruptions on general-, mental- and substance use healthcare services among people with and without HIV in the United States

**DOI:** 10.1186/s12913-025-13690-w

**Published:** 2025-12-29

**Authors:** Jenni M. Wise, Jun Y. Byun, Lorie Benning, Corilyn Ott, Zenoria Causey Pruitt, Sarah Krier, Daniel Lopez, Janet M. Turan, Sheri D. Weiser, Gina Wingood, Tonya Taylor, Jennafer Kwait, Mardge H. Cohen, Brad Aouizerat, Anjali Sharma, Catalina Ramirez, Matthew J. Mimiaga, Lakshmi Goparaju, Anandi N. Sheth, Daniel Merenstein, Valentina Stosor, Adebola Adedimeji, Michael Plankey, Deborah L. Jones, Gypsyamber D’Souza, Tracey Wilson, M. Reuel Friedman, Mirjam-Colette Kempf

**Affiliations:** 1 School of Nursing, University of Alabama at Birmingham, Birmingham, AL USA; 2 Department of Epidemiology, Johns Hopkins Bloomberg School of Public Health, Baltimore, MD USA; 3 School of Medicine, University of Alabama at Birmingham, Birmingham, AL USA; 4 School of Public Health, University of Pittsburgh, Pittsburgh, PA USA; 5 School of Social Work, Boston University, Boston, MA USA; 6 Department of Health Policy and Organization, University of Alabama at Birmingham School of Public Health, Birmingham, AL USA; 7 School of Medicine, University of California at San Francisco, San Francisco, CA USA; 8 Mailman School of Public Health, Columbia University, New York, NY USA; 9 College of Medicine, SUNY Downstate Health Sciences University, Brooklyn, NY USA; 10 Whitman-Walker Institute, Washington, DC USA; 11 Department of Medicine, Stroger Hospital of Cook County, Chicago, IL USA; 12 College of Dentistry, New York University, New York, NY USA; 13 Divisions of General Internal Medicine and Infectious Diseases, Albert Einstein College of Medicine, Bronx, NY USA; 14 School of Medicine, University of North Carolina at Chapel Hill, Chapel Hill, NC USA; 15 Department of Epidemiology, Fielding School of Public Health, University of California Los Angeles, Los Angeles, CA USA; 16 School of Medicine, Georgetown University, Washington, DC USA; 17 Department of Medicine, School of Medicine, Emory University, Atlanta, GA USA; 18 Feinberg School of Medicine, Northwestern University, Evanston, IL USA; 19 Department of Epidemiology & Population Health, Albert Einstein College of Medicine, Bronx, NY USA; 20 Department of Psychiatry and Behavioral Sciences, University of Miami Miller School of Medicine, Miami, FL USA; 21 School of Public Health, SUNY Downstate Health Sciences University, Brooklyn, NY USA; 22 Department of Urban-Global Public Health, School of Public Health, Rutgers University, Newark, NJ USA; 23 School of Public Health, University of Alabama at Birmingham, Birmingham, AL USA; 24 School of Nursing I Family, Community, and Health Systems, University of Alabama at Birmingham, Plaza Building, 212, 2112 11th Ave S, Birmingham, AL 35205 USA

**Keywords:** COVID, Healthcare, HIV, MWCCS, Access, Utilization

## Abstract

**Background:**

Social disruptions (e.g., loss of stable housing, employment, income) during the COVID-19 pandemic have been associated with greater mental healthcare and substance use treatment needs in the general population. People with HIV (PWH) may have experienced greater social disruptions during the COVID-19 pandemic due to pre-existing social vulnerabilities and may have experienced greater interruptions in healthcare during the pandemic due to the co-management of chronic and co-morbid conditions, including mental health and substance use disorder diagnoses, which are prevalent among PWH.

**Methods:**

Between April and September 2020, surveys assessing social disruption and healthcare interruption were conducted among Multicenter AIDS Cohort Study/Women’s Interagency HIV Study Combined Cohort Study participants. Descriptive statistics and multivariable logistic regression models were used to characterize and examine the relationships between social disruptions and interruptions in general, mental health, and substance use care, adjusting for sociodemographic characteristics (age, race and ethnicity, region, income, and employment) and HIV status. Qualitative interviews were conducted to add depth and context to quantitative analysis.

**Results:**

Among 3,665 survey participants (2,238 PWH, 1,427 PWoH), 54% (1214 PWH, 733 PWoH) reported social disruptions and 42% reported healthcare interruptions (972 PWH, 578 PWoH). PWH experiencing ≥ 2 social disruptions had higher odds of missed healthcare appointments (aOR = 1.92, 95%CI:1.56, 2.36) and interrupted mental healthcare (aOR = 2.54, 95%CI:1.83, 3.53) compared to those experiencing < 2. PWoH experiencing ≥ 2 social disruptions had higher odds of missed healthcare appointments, (aOR = 1.65, 95%CI:1.26, 2.17), interrupted mental healthcare (aOR = 2.24, 95%CI:1.38, 3.64), and interrupted substance use treatment (aOR = 2.58, 95%CI:1.15, 5.75) compared to those experiencing < 2. Qualitative interviews elucidated the following reasons for healthcare interruptions: Interruptions in medication and supplies, reduced access to quality healthcare services, self-imposed interruptions related to perceived COVID risk, increased interruptions related to comorbid care needs, reduced access to mental healthcare, and delays in HIV-care.

**Conclusion:**

Healthcare interruptions during the pandemic inequitably affected vulnerable individuals, especially those experiencing greater social disruptions and those with specialized health care needs. Understanding the impact of pandemic related social disruptions on vulnerable individuals provides an opportunity to address disparities and build a more capable health care system.

**Supplementary Information:**

The online version contains supplementary material available at 10.1186/s12913-025-13690-w.

## Introduction

In 2020, amidst the onset of the Coronavirus Disease (COVID-19) pandemic, various social distancing mandates were implemented in the United States (U.S.) to limit the spread of severe acute respiratory syndrome coronavirus 2 (SARS-CoV-2) infection [33]. Among the many systems affected, the U.S. healthcare system encountered numerous challenges, including critical shortages in personnel, medical supplies, personal protective equipment, and space for COVID-19 patients. The Centers for Medicare & Medicaid Services recommended limiting non-emergent, elective, preventive non-COVID-19 healthcare to prioritize services for those who required urgent attention [5]. An abundance of evidence now documents a widespread decrease of healthcare services utilization and delays in diagnostic, routine, and urgent care among the US population during the pandemic [21]. While we know that decreased utilization and delays in care negatively impacted health outcomes and wellbeing of the general population, with continued impact today [34], less is known about the impact of the pandemic on populations particularly vulnerable to changes in our healthcare systems, including those requiring the management of chronic health conditions and those requiring access to specialized care (e.g., mental health care and substance use treatment), such as people with HIV (PWH).

During the pandemic, rapid changes in availability and access to social services and personal resources were experienced by many generating an increase in unmet social needs (e.g., unstable or unaffordable housing, food insecurity, unemployment or loss of financial resources, and lack of affordable childcare) across the U.S. It has been shown, that the increase in unmet social needs, paired with changes in our social connectedness, contributed to an increase in mental health and substance use diagnoses in some while aggravating pre-existing mental health and substance us diagnoses in others [25]. PWH, in particular, may have been disproportionately impacted by social disruptions associated with the pandemic due to pre-existing factors (e.g., social isolation, stigma) influencing socioeconomic vulnerability and mental health outcomes [11, 22, 34, 35]. PWH may have also experienced greater interruptions to healthcare during the pandemic due to HIV-specific care needs and the co-management of chronic, co-morbid conditions, including mental health and substance use disorder diagnoses, which are prevalent among PWH [7]. Understanding the impact of pandemic related social disruptions on various health care services and needs among vulnerable populations provides an opportunity to address widening health disparities generated during the pandemic and may be critical in the development and improvement of systems capable to withstand future public health crises. The purpose of this study was to evaluate the prevalence and impact of healthcare interruptions among men and women living with HIV during the COVID-19 pandemic. The Multicenter AIDS Cohort Study (MACS) and Women’s Interagency HIV Study (WIHS) Combined Cohort Study (MWCCS) was leveraged for its ability to examine the impact of COVID-19 restrictions on healthcare services utilization among PWH and a group of socio-demographically matched seronegative individuals.

## Methods

### Study setting and participants

The Multicenter AIDS Cohort Study (MACS) and Women’s Interagency HIV Study (WIHS) Combined Cohort Study (MWCCS) is a prospective observational cohort study of PWH and a socio-demographically matched group of people at increased behavioral risk for acquiring HIV in the U.S. and aims to understand the impact of chronic health conditions that affect PWH in the U.S [3, 7, 16]. While enrollment criteria has varied across recruitment waves, most (79%) participants are between 40 and 69 years of age, roughly half (53%) are female, and 65% are people of color [7]. Study sites are located at thirteen regional centers across the U.S. While participants routinely attend study visits at biannual intervals which include the collection of socioeconomic, behavioral, and clinical data, MWCCS investigators developed an ancillary COVID-19 survey that assessed social disruption and healthcare interruptions during the pandemic. All participants enrolled in the MWCCS were eligible for this sub-study. Study staff contacted cohort participants via telephone to administer the survey three times, six weeks apart, between April and September 2020. A sub-set of participants were enrolled for qualitative interviews to add depth of understanding related to the impact of social disruptions and healthcare interruptions during the pandemic. This study was approved by the Johns Hopkins Institutional Review Board The research was conducted in accordance with the Declaration of Helsinki. All participants provided informed consent to participate and were offered cash incentives as a token of appreciation.

### Quantitative methods and analysis

The Chronic Care Model was used to inform the research questions and analyses performed [12]. We hypothesized that individual-level social disruptions would be associated with increased interruption in healthcare services during the pandemic and that PWH would be disproportionately impacted by interruptions in care. Individual-level social disruptions were measured using five MWCCS COVID survey items which asked participants if they experienced disruptions (“yes/no”) in in employment, financial support, housing, childcare, or health insurance since the previous study visit [8, 10]. Responses of “yes” were summed and re-categorized as 0, 1, and ≥ 2 to reflect overall social disruption based on previous analysis done within the MWCCS [8, 10].

Interruptions in health care services were measured using the MWCCS COVID-19 survey [8, 10]. At each survey timepoint, participants were asked three “yes/no” questions assessing the prevalence of different types (i.e., general, mental health, and substance use treatment) of healthcare interruptions since the previous timepoint (i.e., “Since the last visit, were you unable to attend a healthcare providers appointment?) Participant’s responding “yes” to any interruption in healthcare were also asked the reason for their interruption (i.e., office closure, transportation issues, affordability issues, or lack of other resources necessary to adapt to available telehealth visit). Those reporting interruptions in specialized care (e.g., mental health and substance use care) were asked about the impact of healthcare interruptions on mental health or substance use care received, with response options of “not at all”, “somewhat” or “a lot”. Responses were then dichotomized to “none at all” and “any” to aid in interpretation. In addition to assessing interrupted healthcare appointments, participants were also asked a single “yes/no” question about their ability to obtain medications they would normally take during the pandemic, with “yes” responses requiring an additional question specifically pertaining to the ability to obtain HIV-medications.

Because we hypothesized that individual level social disruption would be associated with healthcare disruption, we controlled for pre-pandemic sociodemographic factors, including age, sex, race and ethnicity, geographic location, employment and income. Biological sex and race and ethnicity were determined by self-report. Geographic location was assigned based on the participant’s affiliation with the study site (Northeast, Mid-Atlantic, Midwest, West, and Southern United States). Among participants originally enrolled in MACS, employment was assessed by self-report (working full-time, working part time, unemployed, but seeking work, unemployed but not seeking work, student, retired, or disabled) and dichotomized to yes/no. Among participants originally enrolled in WIHS, employment was assessed by asking participants “Are you currently employed?” (yes/no). Income was also assessed based on original enrollment in MACS or WIHS, with former MACS participants being asked to self-report annual income (< $20,000, $20,000 -$39,999; $40,000 - $59,999; ≥$60,000 USD/year) and former WIHS participants being asked to self-report annual household income (≤$6000, $6,001–12,000, $12,001–24,000, $24,001–36,000, 36,001–75,000, or > 75,000 USD/year). Income was then dichotomized based on the criteria for “low income” (<$20,000/year for MACS and ≤ 18,000/year for WIHS). Descriptive statistics were used to characterize and examine differences in sociodemographic factors, social disruptions, and healthcare interruptions. Changes across the three survey waves were stratified by HIV-status and analyzed using two-sided Cochran-Armitage trend tests.

A variable was created to characterize the overall prevalence and impact of healthcare interruptions across the study period by keeping the highest value (e.g., prevalent disruptions, higher impact) reported for each type of health care interruption across survey timepoints. Logistic regression was conducted to test the association between summated social disruptions (0, 1, ≥ 2) and interruption in general, mental health, and substance use health care (*any), adjusting for sociodemographic characteristics. Statistical significance was evaluated at an alpha-level of 0.05. Analyses were performed using SAS 9.4 (SAS Institute, Inc., Cary, NC).

### Qualitative methods and analysis

We conducted 72 in-depth interviews among PWH (*n* = 42, 61.9% female) and PWoH (*n* = 30, 65.5% female) between June-November 2020. Participants who previously completed surveys were eligible to participate in the qualitative interviews. The purpose of the interviews was to understand the impact of the COVID-19 pandemic on: Access to healthcare services; physical and mental health; and psychosocial and economic burden among PLH and PWoH. Study staff at participating sites used purposeful recruitment strategies to generate a diverse sample of participants (*N* = 72) accounting for race and ethnicity (31.5% White non-Hispanic, 57.1% Black non-Hispanic, 6.7% Hispanic any-race, and 5.7% other), gender (63.4% female), financial strain (51.4%), and history of SARS-CoV-2 diagnosis (26.8%) at the time of the interview. Following consent, a team of researchers conducted individual interviews by phone, lasting approximately 30–60 min. Example questions included “Can you tell me about the experiences you’ve had accessing healthcare during the pandemic?”, “How has the COVID-19 pandemic affected your access to medications or basic healthcare supplies?”, “How has your mental health been during the pandemic?”, and “Have you used alcohol or illicit substances to help cope with stressors since the COVID-19 pandemic?” (Supplemental Table 1).

Thematic analysis techniques were utilized to identify, analyze, and report themes emerging from the qualitative data [4, 23]. An initial codebook was created by JW and CO, and tested by DJ and SK to ensure fit across a small batch of transcripts to identify any discrepancies in the meaning and use of codes. Researchers then worked in pairs using inductive and deductive techniques to iteratively review and code the data, until discrepancies between coders had been reconciled. NVivo v14 software was used to facilitate the analysis. Researchers practiced self-reflection related to potential internal biases and preconceptions pertinent to the research to promote rigor [15]. Quantitative and qualitative data analyses for this study occurred independently, with areas of complementarity, convergence, and divergence identified thereafter.

## Results

### Quantitative findings

#### Sociodemographic characteristics, COVID-related social Disruptions, and interruption in healthcare services

A total of 3,665 participants completed surveys for this study, comprising 2,238 PWH (men 39%, women 61%) and 1,427 PWoH (men 60%, women 40%) (Table 1). Participation rates declined across the three timepoints (3,416–3,273), with participation rates remaining consistent by HIV status. Mean age was 56.5 years overall, with PWH being in average younger (55.0 ± 10.2 years) than PWoH (58.7 ± 12.2 years). A high percentage of PWH identified as Black (non-Hispanic ethnicity) (43%), while a high percentage of PWoH identified as White (non-Hispanic ethnicity) (48%). Among PWH, 54% experienced at least one social disruption, and 24% experienced ≥ 2 social disruptions. For PWoH, 51% experienced at least one social disruption, and 21% experienced ≥ 2. Notably, among these social disruptions, a significant difference was found in financial strain with PWH facing greater financial challenges compared to PWoH (23% vs. 16%, *p* < 0.0001).

**Table 1:** Sociodemographic Characteristics, social Disruptions, and interrupted healthcare among men and women with and without HIV

	Overall		PWH		PWoH		*p*-value
	(*N* = 3665)		(*N* = 2238)		(*N* = 1427)		
	*N*	%	*N*	%	*N*	%	
**Sociodemographic characteristics**							
Age in years: Mean (SD)		56.5 (11.2)		55.0 (10.2)		58.7 (12.2)	< 0.0001
Gender							
Men	1737	47	878	39	859	60	
Women	1928	53	1360	61	568	40	
Race and Ethnicity							< 0.0001
White non-Hispanic	1301	36	618	28	683	48	
Black non-Hispanic	1520	41	1046	47	474	33	
Hispanic any race	497	14	351	16	146	10	
Other	347	9	223	10	124	9	
Region of US							< 0.0001
Northeast	539	15	367	16	172	12	
Mid-Atlantic	648	18	372	17	276	19	
Midwest	931	25	532	24	399	28	
West	869	24	473	21	396	28	
South	678	18	494	22	184	13	
Not Employed	1931	53		56		48	< 0.0001
Employed: Annual income (USD before taxes)							
<$20,000/year for MACS, ≤$18,000/year for WIHS	1554	42	1095	49	459	32	
≥$20,000/year for MACS, >$18,000/year for WIHS	2110	58	1142	51	968	68	< 0.0001
**Social disruptions**							
0	1718	47	1024	46	694	49	
1	711	19	453	20	258	18	
≥ 2	1236	34	761	34	475	33	0.03
**Interruptions in Healthcare**							
Unable to attend a healthcare providers appointment	1550	42	972	43	578	41	0.08
Number of times reported unable to attend a healthcare providers appointment							0.19
0	2115	58	1266	57	849	59	
1	1036	28	650	29	386	27	
2	385	11	235	10	150	11	
3	129	4	87	4	42	3	
→ If yes, was it because…							
- Provider’s office was closed due to coronavirus pandemic	1250	81	786	81	464	80	0.77
- Didn’t have transportation	119	8	84	9	35	6	0.07
- Provider was seeing patients online (no internet) or by phone (no phone)	440	29	284	29	156	27	0.37
Unable to obtain medications that you normally take	302	8	190	8	112	8	0.49
→ If yes, were these your HIV medications? (Y)	121	64	121	64			
Unable to afford medical care	142	4	79	4	63	4	0.18
Receive mental healthcare	1364	37	908	41	456	32	< 0.0001
How much has the coronavirus pandemic interrupted the care you receive for mental health?							0.01
Not at all	648	48	435	48	213	47	
Somewhat	365	27	221	24	144	32	
A lot	351	36	252	28	99	22	
Receive substance use addiction care	522	14	352	16	170	12	0.001
How much has the coronavirus pandemic interrupted the care you receive for substance use addiction?							0.79
Not at all	335	64	229	65	106	62	
Somewhat	105	20	68	19	37	22	
A lot	82	16	55	16	27	16	

Note. PWH indicate PWH. PWoH indicate PWoH. Wave 1 (April - May); Wave 2 (June - July); and Wave 3 (August - September). Northeast (Brooklyn NY, Bronx NY), Mid-Atlantic (Washington DC, Baltimore MD), Midwest (Chicago IL, Pittsburgh PA, Columbus OH), West (San Francisco CA, Los Angeles CA), South (Chapel Hill NC, Atlanta GA, Miami FL, Birmingham AL, Jackson MS)

While 42% of participants reported missing at least one healthcare providers appointment, no difference was detected by HIV-status. Few (8%) participants reported interruption in the ability to access medications they would normally take. While no differences in any medication interruption were present by HIV status (*p* = 0.49), 64% of PWH difficulty accessing the necessary medication to manage their HIV-infection. Overall, 37% (*n* = 1,356) of participants reported receiving mental healthcare prior to the pandemic, with PWH being more likely to report mental health care treatment compared to PWoH.

(41% vs. 32%, *p* < 0.0001). Nearly two-thirds (63%) of participants reporting mental healthcare service prior to the pandemic reported interruption in mental healthcare, with PWH being more likely to report “a lot” of negative impact of mental healthcare interruptions compared to PWoH (28% vs. 22%, *p* = 0.01). While 14% of participants (*n* = 513) reported receiving substance use care prior to the pandemic, PWH were more likely to report prior engagement in substance use care (16% vs. 12%, *p* = 0.001). Roughly a third (36%) of participants reported interruptions in substance use treatment, with no difference detected by HIV-status (*p* = 0.79).

#### Interrupted healthcare services by HIV status and survey wave

**Fig. 1:**
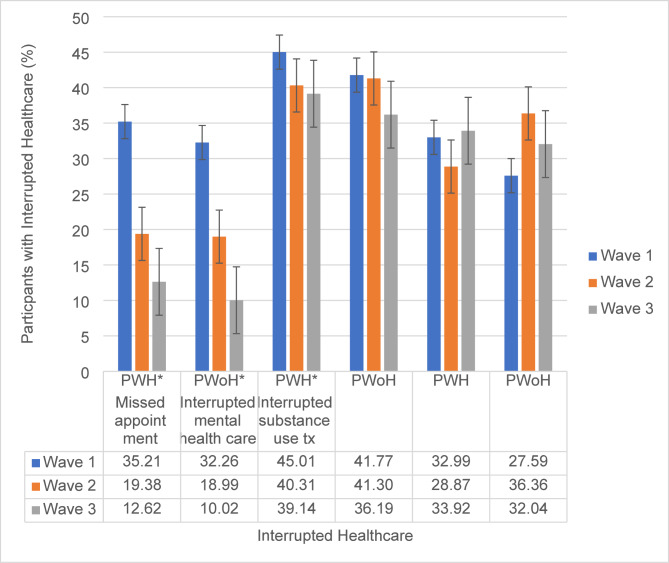
Interrupted healthcare by HIV status and survey wave. Note. PWH indicate PWH. PWoH indicate PWoH. Wave 1 (April – May 2020); Wave 2 (June – July 2020); and Wave 3 (August-September 2020). * *P-*value less than 0.05

In total, 3665 participants completed at least one survey over the course of the study with 7%, (66.5% PWH) 12%, (64.3% PWH) and 82% (60.2%) of participants completing one, two, or three COVID-19 surveys respectively. Figure 1 illustrates changes in interrupted healthcare based on HIV status and survey wave. Among PWH, there was a decline in missed healthcare appointments across time (wave 1 = 35.2%, wave 2 = 19.4%, wave 3 = 12.6%, p-*trend* < 0.0001), accompanied by a decrease in interrupted mental healthcare (wave 1 = 45.0%, wave 2 = 40.3%, wave 3 = 39.1%, p-*trend* = 0.035) albeit mental health was interrupted at a much higher rate and less change over time. There was no significant change in interrupted substance use treatments over time among PWH (wave 1 = 33.0%, wave 2 = 28.9%, wave 3 = 33.9%, p-*trend* = 0.88). Among PWoH, missed healthcare appointments decreased over time (wave 1 = 32.3%, wave 2 = 19.0%, wave 3 = 10.0%, p-*trend* < 0.0001), while there was no significant change in interrupted mental healthcare (wave 1 = 41.8%, wave 2 = 41.3%, wave 3 = 36.2%, p-*trend* = 0.15) and interrupted substance use treatment (wave 1 = 27.6%, wave 2 = 36.4%, wave 3 = 32.0%, p-*trend* = 0.54).

#### Associations between social disruptions and interrupted healthcare services

Table 2 summarizes results from multivariate analyses assessing the effects of social disruptions on healthcare services interruptions. In adjusted models, experiencing one social disruption was significantly associated with higher odds of missed healthcare appointments (aOR = 1.51; 95%CI:1.20–1.90) and interrupted mental healthcare (aOR = 1.60; 95%CI:1.11–2.30) among PWH. Furthermore, the likelihood of missing healthcare appointments (aOR = 1.92; 95%CI:1.56–2.36) and experiencing interrupted mental healthcare (aOR = 2.54; 95%CI:1.83–3.53) increased among those experiencing 2$\:\ge\:$ social disruptions. PWH were more likely to report “a lot” negative impact on their mental healthcare needs (aOR=2.96, 95% CI: 2.05–4.29) (data not shown). However, social disruptions did not show any significant association with interruptions in substance use treatment among PWH.

**Table 2:** Associations between extent of social disruptions and interrupted healthcare in the MWCCS: results from multivariate logistic regressions

	Multivariate models								
	Missed appointment			Interrupted mental healthcare (any)*			Interrupted substance use tx (any)**		
	PWH (2238)	PWoH (1427)	Overall (3665)	PWH (908)	PWoH (456)	Overall (1364)	PWH (352)	PWoH (170)	Overall (522)
Social disruptions									
0 (reference)									
1	**1.51** **(1.20–1.90)**	1.08 (0.79–1.47)	**1.33** **(1.11–1.60)**	**1.60** **(1.11–2.30)**	**1.88** **(1.11–3.19)**	**1.64** **(1.22–2.21)**	1.41 (0.76–2.62)	**2.80** **(1.09–7.17)**	**1.72** **(1.03–2.86)**
2+	**1.92** **(1.56–2.36)**	**1.65** **(1.26–2.17)**	**1.81** **(1.54–2.13)**	**2.54** **(1.83–3.53)**	**2.24** **(1.38–3.64)**	**2.42** **(1.85–3.17)**	1.70 (0.98–2.95)	**2.58** **(1.15–5.75)**	**1.97** **(1.26–3.09)**
Age									
≥60 (reference)									
<40	1.03 (0.72–1.47)	0.70 (0.44–1.12)	0.89 (0.67–1.18)	1.34 (0.77–2.33)	2.06 (0.91–4.69)	1.46 (0.93–2.29)	1.66 (0.57–4.83)	0.50 (0.07–3.50)	1.22 (0.49–3.03)
40–49	1.16 (0.89–1.51)	0.95 (0.65–1.39)	1.08 (0.87–1.34)	1.21 (0.80–1.81)	1.48 (0.77–2.85)	1.18 (0.84–1.66)	1.40 (0.67–2.90)	0.89 (0.30–2.66)	1.15 (0.64–2.06)
50–59	1.25 (1.01–1.54)	1.15 (0.85–1.56)	**1.21** **(1.02–1.43)**	1.33 (0.95–1.86)	1.16 (0.69–1.95)	1.27 (0.96–1.68)	1.35 (0.77–2.38)	1.70 (0.71–4.03)	1.44 (0.91–2.29)
Race and Ethnicity									
White non-Hispanic (reference)									
Black non-Hispanic	**0.76** **(0.59–0.98)**	0.81 (0.57–1.13)	**0.78** **(0.64–0.95)**	0.87 (0.59–1.30)	0.92 (0.52–1.62)	0.87 (0.63–1.20)	0.55 (0.28–1.09)	0.95 (0.34–2.67)	0.70 (0.40–1.22)
Hispanic any race	0.92 (0.68–1.24)	1.03 (0.67–1.60)	0.95 (0.75–1.22)	0.84 (0.51–1.37)	0.68 (0.32–1.44)	0.79 (0.53–1.18)	0.40 (0.15–1.05)	0.68 (0.14–3.34)	0.51 (0.23–1.15)
Other	0.90 (0.63–1.27)	1.07 (0.67–1.72)	0.95 (0.72–1.25)	1.01 (0.61–1.68)	1.03 (0.50–2.12)	0.97 (0.64–1.47)	**0.40** **(0.16–0.97)**	0.53 (0.14–1.99)	**0.46** **(0.22–0.96)**
Region									
West (reference)									
Northeast	**1.92** **(1.39–2.67)**	**1.98** **(1.25–3.13)**	**1.94** **(1.49–2.52)**	0.80 (0.48–1.32)	1.83 (0.80–4.19)	0.99 (0.65–1.51)	0.44 (0.15–1.30)	0.22 (0.05–1.02)	**0.36** **(0.15–0.83)**
Mid-Atlantic	**1.48** **(1.11–1.98)**	**1.62** **(1.16–2.26)**	**1.55** **(1.24–1.92)**	1.07 (0.68–1.69)	1.45 (0.80–2.61)	1.18 (0.83–1.69)	0.78 (0.36–1.72)	**0.21** **(0.06–0.71)**	**0.51** **(0.27–0.97)**
Midwest	**1.80** **(1.38–2.34)**	**1.89** **(1.40–2.56)**	**1.85** **(1.52–2.26)**	**1.71** **(1.09–2.68)**	0.99 (0.56–1.75)	**1.43** **(1.01–2.02)**	1.75 (0.80–3.86)	0.44 (0.16–1.24)	1.07 (0.58–1.95)
South	**1.78** **(1.29–2.44)**	**2.28** **(1.44–3.61)**	**1.94** **(1.50–2.51)**	0.76 (0.48–1.22)	1.97 (1.00-3.88)	1.00 (0.69–1.47)	**0.40** **(0.17–0.95)**	**0.30** **(0.09–0.97)**	**0.37** **(0.19–0.71)**
Not employed	**1.40** **(1.14–1.71)**	**1.53** **(1.18–1.97)**	**1.46** **(1.25–1.71)**	**1.93** **(1.37–2.73)**	**2.22** **(1.38–3.57)**	**2.02** **(1.54–2.66)**	1.00 (0.55–1.79)	0.87 (0.38–1.99)	0.96 (0.60–1.52)
Low income	1.08 (0.88–1.32)	1.30 (0.98–1.73)	1.13 (0.96–1.34)	1.13 (0.81–1.56)	1.26 (0.75–2.09)	1.11 (0.85–1.46)	1.63 (0.92–2.91)	1.58 (0.66–3.74)	1.47 (0.93–2.35)
WIHS vs. MACS	1.07 (0.83–1.38)	1.13 (0.76–1.70)		1.12 (0.76–1.66)	0.54 (0.29–1.01)		0.97 (0.50–1.85)	0.91 (0.31–2.69)	

Note. PWH indicate PWH. PWoH indicate PWoH. WIHS indicates Women’s Interagency HIV Study. MACS indicates the Multicenter AIDS Cohort Study. Low income indicates <$20,000/year for MACS and ≤$18,000/year for WIHS. Northeast (Brooklyn NY, Bronx NY), Mid-Atlantic (Washington DC, Baltimore MD), Midwest (Chicago IL, Pittsburgh PA, Columbus OH), West (San Francisco CA), South (Chapel Hill NC, Atlanta GA, Miami FL, Birmingham AL, Jackson MS)

* Experiencing interruptions ‘somewhat’ or ‘a lot’ in mental healthcare

** Experiencing interruptions ‘somewhat’ or ‘a lot’ in substance use treatment

Differences in interrupted healthcare services were observed among PWH by race and ethnicity, employment status, and region of residence. We found that Black (non-Hispanic) participants were less likely to experience healthcare interruptions in general (aOR = 0.76; 95%CI:0.59–0.98). PWH without employment had higher odds of missed healthcare appointments (aOR = 1.40; 95%CI:1.14–1.71) and interrupted mental healthcare (aOR = 1.88; 95%CI:1.11–3.19) compared to those employed. PWH in the Northeast were more likely to experience interruptions in general healthcare (aOR = 1.92, 95%CI:1.39, 2.67) compared to other U.S. regions.

Among PWoH, those with one social disruption showed higher odds of interrupted mental healthcare (aOR = 1.88; 95%CI:1.11–3.19) and interrupted substance use treatment (aOR = 2.80; 95%CI:1.09–7.17) but not missed healthcare appointments, while those experiencing $\:\ge\:$2 disruptions had higher odds of missed healthcare appointments (aOR=1.65; 95%CI:1.26–2.17), interrupted mental healthcare (aOR=2.24; 95%CI:1.38–3.64), and interrupted substance use treatment (aOR = 2.58; 95%CI:1.15–5.75). PWoH who reported unemployment exhibited higher odds of missed healthcare appointments (aOR=1.53; 95%CI:1.18–1.97) and interrupted mental healthcare (aOR=2.22; 95%CI:1.38–3.57). PWoH in the South were more likely (aOR = 2.28, 95% CI: 1.44, 3.61) to experience interruptions in general care compared to other US regions, and PWoH were more likely to experience interruptions in general care compared to PWH. Bivariate associations between social disruptions and interrupted healthcare services are shown in Supplemental Table 2.

### Qualitative findings

Interview participants were mostly female (63.8%) with a mean age of 52 years (Table 3). Nineteen (26.8%) reported a history of SARS-Cov2 infection at the time of the interview. Over half (61%) reported changes in healthcare access including restrictions to in-person visits, changes in staffing and procedures impacting quality of care, cancellation of primary and specialty care appointments, and general dissatisfaction with adaptations made to maintain healthcare services. Key themes emerging from the data included: (1) interruptions in medications and supplies, (2) quality of healthcare services and reduced access, (3) self-imposed interruptions in healthcare related to COVID risk, (4) increased co-morbidity related healthcare services interruptions, (5) unmet mental health care needs, and (6) interruptions in HIV-care.

**Table 3:** Descriptive characteristics of MWCCS Participants – Qualitative interviews

	PWH	PWoH	Total	
**Demographic Characteristics**				
Age (years), mean (SD)	53.2 (9.3)	51.7 (12.0)	52.6 (10.4)	
Race and Ethnicity				
White non-Hispanic	15 (35.7)	7 (25)	22 (31.5)	
Black non-Hispanic	22 (52.4)	18 (64.3)	40 (57.1)	
Hispanic any race	2 (4.8)	2 (7.1)	4 (5.7)	
Other	3 (7.1)	1 (3.6)	4 (5.7)	
Sex				
Male	16 (38.1)	10 (34.5)	26 (36.6)	
Female	26 (61.9)	20 (65.5)	45 (63.4)	
History of COVID-19^a^	11 (26.2)	8 (27.6)	19 (26.8)	
Financial Strain^b^	21 (50)	15 (53.6)	36 (51.4)	
Region of US Country^c^				
Northeast (Brooklyn NY, Bronx, NY)	7 (16.7)	4 (13.8)	11 (15.5)	
Mid-Atlantic (Washington DC, Baltimore MD)	7 (16.7)	5 (17.2)	12 (16.9)	
Midwest (Chicago IL, Pittsburgh, PA, Columbus, OH)	11 (26.2)	7 (24.1)	18 (25.4)	
West (San Francisco CA)	3 (7.1)	2 (6.9)	5 (7.0)	
South (Chapel Hill NC, Atlanta GA, Miami FL, Birmingham AL, Jackson MS)	14 (33.3)	11 (37.9)	25 (35.2)	
**Socioeconomic Characteristics**				
Income^d^				
≤ 18,000 USD /year	17 (40.5)	11 (30.3)	28 (40)	
> 18,000 USD/year	25 (59.5)	17 (60.7)	42 (60)	
Employed				
Full-time	13 (31.0)	10 (35.7)	23 (32.9)	
Part-time	5 (11.9)	6 (21.4)	11 (15.8)	
Retired	3 (7.1)	5 (17.9)	8 (11.4)	
Disability	12 (28.6)	3 (10.7)	15 (21.4)	
Insurance^e^	39 (92.9)	26 (92.8)	65 (92.9)	

^a^Self-reported history of COVID-19 at time of interview

^b^Self reported challenges paying for basic needs such as food, clothing, shelter, and heating

^c^Participant affiliation with MWCCS site

^d^ Household income/year

^e^Health insurance, ADAP, or Ryan White

#### Interruptions in medication and supplies

Participants described how COVID-19 related social disruptions negatively impacted their effective routines in securing medications and medical supplies necessary to effectively manage chronic health conditions. This was attributed to a variety of factors, including excessive strain on the healthcare system and changes in clinic and system-level procedures - leaving gaps in previously effective healthcare services.

Every so often, the supply company calls me to see if I need supplies. When I tell them what I need, “Well, we gotta get your doctor’s approval.” They haven’t been able to get his approval because the rules are I have to see the doctor in order for him to approve everything. -White male without HIV

Interruptions in supply chains were often attributed to breakdowns in communication and shifts in role responsibilities. Co-occurring strain on other social systems and services (e.g., changes in insurance, case management, and postal system) was mentioned as impacting the availability of medications and medical supplies.

I had around a four-day period where I didn’t have my medication, because I’m waitin’ for it to come in the mail….I just think it might’ve just been difficult at moments for people with HIV, on medication and just keepin’ our healthcare consistent…. It’s been a stressful situation. -White male with HIV

#### Quality of healthcare services and reduced access

While some participants expressed satisfaction with adaptations made to healthcare services, like the flexibility of telehealth, concerns frequently arose related to the quality of healthcare services available. Some participants described technological barriers with limited or incompatible access to smart phones or computers necessary for telehealth visits.

The only problem I’m havin’ is I have to call my doc. I have to deal with my doctor through the phone. I have one doctor that I just can’t seem to deal with because they only do it with computers, and I only have a phone. -White male without HIV

While most participants were able to participate in telehealth visits, concerns frequently described included innate limitations in the conduct of physical exams and communication challenges during visits, including the ability to establish rapport with providers and adequately convey healthcare needs. Individuals receiving mental healthcare were more likely to be dissatisfied with the transition to telehealth visits.

*Every doctor’s visit and psychiatrist and everybody is on the phone. Healthcare is not through a phone. It’s through being able to show ’em what’s goin’ on*,* and I haven’t been able to really do* that….it’s been quite difficult. -Black female with HIV

#### Self-imposed interruptions in healthcare related to COVID-19 risk

Concerns related to SARS-Cov2 exposure and transmission commonly appeared as a driving force behind participants’ decisions to abstain from healthcare services during the pandemic. This was particularly the case for PWH or PWoH with chronic diseases such as heart disease and diabetes, with common concerns including greater vulnerability to severe complications and/or mortality.

I canceled dentist appointments because I did not wanna…have somebody standin’ over me who might’ve possibly had COVID…. I canceled my eye appointment. Didn’t want anybody lookin’ in my eyes, and they got COVID or don’t even know they have COVID. I canceled my annual doctor’s….That was just the beginning. -White female without HIV

I don’t really feel safe, so I don’t really go unless it’s really necessary. -Black female without HIV

##### Increased co-morbidity related healthcare services interruptions

Participants with chronic and co-morbid health conditions existing prior to the pandemic described greater interruptions of routine healthcare services during the pandemic, including the delay or cancellation of what they considered to be medically necessary procedures.

I am dealing with a longstanding problem…. Because of COVID, I wasn’t able to go into the office. I’ve delayed dealing with it. …. I have a new one now that I need to deal with. -Black male with HIV

Limited access to routine screening and preventative services important to the management and treatment of chronic diseases were common experiences. While PWH without other chronic health management needs reported similar interruptions in healthcare compared to PWoH, PWH with co-morbid, chronic conditions described greater interruptions in healthcare services.

I was going in on March 17th for heart surgery which was cancelled. …. All of my medical providers’ appointments are cancelled. My cardiac rehab was cancelled. That was the medical part of it……That’s jerking you around a little bit too…. It’s emotionally stressful. -White female with HIV

#### Unmet mental healthcare needs

Although most participants experienced some sort of interruption to healthcare services received prior to the pandemic, those who were previously receiving mental health services before the pandemic were more likely to describe a negative impact of COVID-19 related social disruptions on mental health. Participants with mental healthcare interruptions described an inability to access mental healthcare during the pandemic and/or dissatisfaction with the quality of care received.

Right in the middle of the pandemic, my mental health provider was gettin’ ready to leave the clinic and move back to her hometown…. that’s not too cool to go from seein’ somebody once a week to talk about your problems and stuff, to not seein’ ’em at all. That has taken a big toll on me, the mental health part. -Black female with HIV

The negative impact of pandemic-related stressors on mental health commonly characterized the stories of all participants (e.g., both those receiving mental health services before the pandemic and those with mental healthcare needs arising during the pandemic) with shared themes of experiencing depression, anxiety, stress, and loneliness. PWH were more likely to describe greater unmet mental healthcare needs and particular stress related to re-traumatization related to the uncertainty and fear associated with the pandemic.

Because of COVID, my counseling therapy session’s been canceled too. I really have a lot of issues with that. I don’t talk to no one right now, because it’s not one to that, but it’s been an issue, and it’s been really scary, but hopefully, I get back to normal soon. -Hispanic female with HIV

While quantitative results indicate that 14% of participants experienced interruptions in substance use treatment during the pandemic, none of the participants completing interviews described explicit interruption in substance use-related care. However, participants commonly shared stories of heightened stress and uncertainty during the pandemic with some participants describing maladaptive coping mechanisms - including the increased use of marijuana and alcohol.

##### Interruptions in HIV-care

While most PWH participants were able to continue their HIV care via telehealth and modified delivery of healthcare services, participants described challenges accessing medications and expressed limitations inherent to remote health visits, including the lack of routine lab work necessary for the management of ART and co-morbidities common with HIV-infection.

I haven’t even had blood drawn since the beginning of the year. I don’t know what’s going on in that respect. I’m only doing what they’re asking me to do by takin’ my medicine all the time, hopin’ … that’ll be just fine. -Black male with HIV

Participants described how the impact on HIV care occurred early in the pandemic, with resolution occurring over several weeks.

I’ve been tryin’ to call [my doctor], but they haven’t answered……about my depression pills and my HIV pills. I need a prescription, ’cause I hadn’t had it in a while to get it. -Indian female with HIV

## Discussion

While interruptions to general healthcare decreased over time, interruptions to specialized care (i.e., mental health care and substance use treatment) remained high during the study period (April – September 2020). People who were more vulnerable to social disruptions during the pandemic were also more likely to require specialty care related to pre-existing health care conditions as well as emerging needs exacerbated by the pandemic. Interruptions to care disproportionately and negatively impacted people in need of mental health and substance use care. While PWH were disproportionately and negatively impacted by mental healthcare interruptions, PwoH were more vulnerable to interruptions in substance use care. Our qualitative data also indicated that individuals with more complex healthcare needs (i.e., individuals with chronic or comorbid conditions) and those requiring treatment less amendable to telehealth adaptations faced particular challenges regarding healthcare interruptions during the pandemic. Notably, disruptions to HIV-specific care were minimal, with 8% PWH reporting issues accessing HIV-medication during the pandemic.

Overall, these findings support the existing literature that the COVID-19 pandemic impacted access to healthcare services and that while individual and system level adaptations decreased the impact to general healthcare services relatively quickly, they were not sufficient to address the pre-existing and widening healthcare needs of those requiring more specialized care- including those requiring mental health care and substance use treatment [19, 29, 36]. Additionally, we know that while the heightened levels of stress associated with the pandemic impacted mental health broadly across the nation, the mental health burden was more pronounced among certain populations, including individuals experiencing or at risk for greater social disruptions (e.g., homelessness, job loss) and those at increased risk for adverse mental health outcomes (e.g., those with low social support) prior to the pandemic [10, 25, 27]. Nationwide, pandemic related disruptions also coincided with an increase in reported substance and alcohol use, including increases in drug-overdoses and alcohol induced deaths- highlighting the need for more resilience of specialty care services [25]. 

It is now evident that population level social disruptions magnified with underlying risk for social disruptions at the individual and household level, places populations who have less socioeconomic resources (including women and people of color) [14, 18] at increased risk for personal and economic instability during a public health crisis while simultaneously placing them at heightened need for care [23, 24, 26, 31]. This pre-existing social vulnerability prior to the pandemic likely widened existing health disparities highlighted within our study – such as those related to mental health and substance use care [6, 20]. Among the general population, it is now estimated that depression and anxiety rose from 10% (pre-pandemic) to 20–35% over the course of the pandemic [9, 24, 28]. Considering that PWH are two to four times more likely to develop depression and anxiety compared to the general population, the impact of the pandemic on the mental health of PWH becomes of particular concern [32]. Similarly, we know that drug-overdose death rates rose 50% during the pandemic, and that individuals with HIV and people at behavioral risk of acquiring HIV (such as those in our cohort) are much more likely to have substance use healthcare needs compared to the general population [25]. Whereas this study was not designed to examine changes in mental healthcare and substance use needs during the pandemic, other studies demonstrate an increase in mental health and substance abuse needs among PWH during the pandemic [1, 2, 13, 17, 26, 30, 31]. Multi-level interventions are needed to address the causes of population and individual level social factors contributing to the negative impact of public health or natural disasters and build healthcare systems more resilient to withstand the breakdown of healthcare services in times of distress with particular attention and priority given to vulnerable populations at high risk.

### Strengths and limitations

While this study has strengths including a large sample size demographically representative of PWH and sociodemographically matched PWoH in the U.S., limitations exist. Namely, the data was self-reported by participants and may be biased due to under- or over-reporting of pre-existing healthcare needs as well as the impact of healthcare interruptions. While the study’s sample population for quantitative data was fairly balanced by sex, the sample for qualitative data had a higher proportion of female participants, and thus, may not be generalizable to the entire population. We also recognize that PWH within the MACS/WIHS cohorts may have access to medical and social services at a disproportionately higher rate than the general population of PWH in the US. Results should be interpreted cautiously as they may underestimate the overall impact of COVID-19 social disruptions and healthcare interruptions among the general population of PWH and PWoH in the US. Moreover, we recognize that our results may be biased in that individuals experiencing the most severe social disruptions during the pandemic may have been inaccessible to complete these ancillary surveys related to displacement or loss of normal resources used for communication (e.g., phone service). While our research focused on the impact of social disruptions on healthcare services, our study was not designed to detect the increased demands in healthcare services during the pandemic or in the period past September 2020 or the impact of pandemic-related social disruptions on HIV-preventative and/or testing services. Moreover, our study was not designed to examine the impact of COVID on social disruptions and/or healthcare interruptions outside of the U.S.

## Conclusion

The COVID-19 pandemic exacerbated existing health disparities and created new healthcare challenges, particularly among those experiencing socioeconomic vulnerabilities, chronic or co-morbid conditions, and pre-existing mental health or substance use needs. Considering the overall increase and long-lasting impact of unmet healthcare needs and the current incapacity of our healthcare system to effectively meet the healthcare needs during times of distress, strategies must be developed to effectively mitigate existing disparities and create a healthcare system more resilient to future insults. Furthermore, strategies are needed to address social inequities influencing healthcare needs and access across all populations. Sustained efforts to expand and maintain access to quality healthcare are needed, particularly among those who are vulnerable to socioeconomic inequities or have underlying mental health and substance use needs. Investments in public health infrastructures and specialty care systems are urgently needed to prepare them and communities for public health crises in the future.

## Supplementary Information

Below is the link to the electronic supplementary material.

Supplementary Material 1

Supplementary Material 2

## Data Availability

Access to individual-level data from the MACS/WIHS Combined Cohort Study Data (MWCCS) may be obtained upon review and approval of a MWCCS concept sheet.  Links and instructions for online concept sheet submission are on the study website.
