# Supplementary material for: The impact of COVID-19 social disruptions on general-, mental- and substance use healthcare services among people with and without HIV in the United States
Source: BMC Health Serv Res. 2025 Dec 29;25:1623. doi: 10.1186/s12913-025-13690-w (PMC12751715; doi:10.1186/s12913-025-13690-w)
Supplement: Supplementary file 1 — Supplementary Material 1 [file 12913_2025_13690_MOESM1_ESM.docx]

| **Healthcare Utilization** |  |
| --- | --- |
| 1. Can you tell me about any experiences you have had accessing any type of health care during the COVID-19 epidemic? |  |
| 1. How do you feel about going to a clinic or doctor’s office during this time? |  |
| 1. Can you tell me about any times you needed healthcare related to any COVID-19 related symptoms? |  |
| **Mental Health** | |
| 1. How has your mental health been during the COVID-19 epidemic? | |
| 1. How much fear or worry do you experience about getting COVID-19? | |
| 1. What kind of things can trigger you to think about COVID-19, or cause worry about being infected? | |
| 1. Can you tell me about things you might be doing to help you cope with stressors since the COVID-19 pandemic? Have you used any of the following: meditation, exercise, gardening, alcohol, or illicit substance use? | |
| **Social Support** | |
| 1. How has COVID-19 affected your social life? | |
| 1. What do you do to maintain your social connections? | |
| 1. Who are the people you are relying the most on for support during the COVID-19 epidemic? | |
| 1. How have shelter-in-place or stay at home orders affected your relationships with partners or family? | |
| 1. How do you think people who have the COVID-19 virus or who are suspected to have the virus are perceived by others? | |
| 1. How do you think people living with HIV are affected by the COVID-19 epidemic? | |
| **Other Impact** | |
| 1. Are there any other ways that the COVID-19 epidemic has affected you, such as: employment, income, housing, access to other basic supplies, physical activity, diet, and access to medications? | |

Supplemental Table 1. Interview items related to healthcare disruptions among MWCCS participants during the COVID-19 pandemic.
