# Supplementary material for: The impact of COVID-19 social disruptions on general-, mental- and substance use healthcare services among people with and without HIV in the United States
Source: BMC Health Serv Res. 2025 Dec 29;25:1623. doi: 10.1186/s12913-025-13690-w (PMC12751715; doi:10.1186/s12913-025-13690-w)
Supplement: Supplementary file 2 — Supplementary Material 2 [file 12913_2025_13690_MOESM2_ESM.docx]

**Supplement Table 2. Associations Between Extent of Social Disruptions and Interrupted Healthcare in the MWCCS: Results From Bivariate Regressions**

|  | **Bivariate models** | | | | | | | | |
| --- | --- | --- | --- | --- | --- | --- | --- | --- | --- |
|  | Missed appointment | | | Interrupted mental healthcare (any)* | | | Interrupted substance use tx (any)** | | |
|  | PWH (2238) | PWoH (1427) | Overall (3665) | PWH  (908) | PWoH (456) | Overall (1364) | PWH  (352) | PWoH (170) | Overall (522) |
| Social disruptions |  |  |  |  |  |  |  |  |  |
| 0 (reference) |  |  |  |  |  |  |  |  |  |
| 1 | **1.48**  **(1.19-1.86)** | 1.07  (0.79-1.43) | **1.32**  **(1.10-1.57)** | **1.55**  **(1.09-2.20)** | **1.67**  **(1.02-2.74)** | **1.58**  **(1.19-2.10)** | 1.40  (0.78-2.53) | **2.55**  **(1.07-6.09)** | **1.69**  **(1.04-2.74)** |
| 2+ | **1.76**  **(1.45-2.13)** | **1.51**  **(1.19-1.92)** | **1.66**  **(1.43-1.93)** | **2.19**  **(1.62-2.96)** | **1.86**  **(1.22-2.84)** | **2.07**  **(1.62-2.65)** | **1.74**  **(1.05-2.88)** | **2.55**  **(1.25-5.23)** | **1.98**  **(1.31-2.98)** |
| Age |  |  |  |  |  |  |  |  |  |
| ≥60 (reference) |  |  |  |  |  |  |  |  |  |
| <40 | 1.16  (0.84-1.60) | 1.00  (0.67-1.49) | 1.10  (0.86-1.41) | 1.31  (0.80-2.17) | 1.73  (0.87-3.43) | 1.44  (0.96-2.15) | 1.01  (0.40-2.59) | 0.82  (0.15-4.56) | 0.97  (0.43-2.19) |
| 40-49 | **1.30**  **(1.03-1.66)** | 1.21  (0.89-1.66) | **1.29**  **(1.07-1.55)** | **1.16**  **(0.80-1.69)** | 1.27  (0.75-2.13) | 1.19  (0.88-1.60) | 1.01  (0.54-1.91) | 1.27  (0.54-2.99) | 1.09  (0.66-1.81) |
| 50-59 | **1.33**  **(1.09-1.62)** | **1.41**  **(1.09-1.82)** | **1.37**  **(1.18-1.60)** | **1.36**  **(0.99-1.87)** | 1.19  (0.77-1.84) | **1.30**  **(1.01-1.68)** | 1.21  (0.72-2.04) | 1.58  (0.77-3.23) | 1.31  (0.86-2.00) |
| Race and Ethnicity | |  |  |  |  |  |  |  |  |
| White non-Hispanic (reference) | |  |  |  |  |  |  |  |  |
| Black  non-Hispanic | 1.06  (0.87-1.30) | **1.32**  **(1.04-1.68)** | **1.19**  **(1.02-1.38)** | 0.91  (0.65-1.28) | **1.20**  **(0.78-1.84)** | 1.00  (0.77-1.30) | **0.54**  **(0.29-0.98)** | 1.03  (0.47-2.27) | 0.68  (0.42-1.10) |
| Hispanic  any race | 1.11  (0.85-1.44) | 1.24  (0.86-1.78) | 1.20  (0.98-1.48) | 0.86  (0.56-1.33) | 0.88  (0.47-1.64) | 0.88  (0.62-1.25) | 0.44  (0.19-1.05) | 0.94  (0.28-3.17) | 0.58  (0.29-1.16) |
| Other | 1.19  (0.87-1.62) | **1.61**  **(1.10-2.37)** | **1.37**  **(1.08-1.74)** | 1.05  (0.67-1.64) | **1.16**  **(0.64-2.11)** | 1.09  (0.76-1.56) | **0.39**  **(0.18-0.86)** | 0.66  (0.23-1.92) | **0.48**  **(0.25-0.90)** |
| Region |  |  |  |  |  |  |  |  |  |
| West (reference) | |  |  |  |  |  |  |  |  |
| Northeast | **1.84**  **(1.39-2.43)** | **2.30**  **(1.59-3.33)** | **2.02**  **(1.62-2.52)** | 0.79  (0.51-1.22) | 1.41  (0.71-2.80) | 0.93  (0.64-1.33) | 0.38  (0.14-1.03) | 0.32  (0.10-1.00) | **0.35**  **(0.16-0.74)** |
| Mid-Atlantic | **1.42**  **(1.08-1.89)** | **1.55**  **(1.12-2.15)** | **1.48**  **(1.20-1.83)** | 1.03  (0.67-1.59) | 1.13  (0.66-1.95) | 1.07  (0.77-1.50) | 0.74  (0.35-1.55) | **0.24**  **(0.08-0.74)** | **0.51**  **(0.28-0.93)** |
| Midwest | **1.71**  **(1.33-2.22)** | **1.83**  **(1.36-2.45)** | **1.77**  **(1.46-2.15)** | **1.60**  **(1.04-2.44)** | 1.11  (0.65-1.88) | **1.40**  **(1.01-1.95)** | 1.90  (0.90-4.01) | 0.67  (0.27-1.63) | 1.24  (0.70-2.19) |
| South | **1.84**  **(1.42-2.38)** | **2.75**  **(1.92-3.95)** | **2.12**  **(1.72-2.61)** | 0.93  (0.63-1.37) | **1.89**  **(1.09-3.27)** | 1.15  (0.84-1.57) | 0.50  (0.25-1.00) | 0.41  (0.16-1.02) | **0.44**  **(0.25-0.75)** |
| Not employed | **1.26**  **(1.06-1.49)** | **1.54**  **(1.24-1.90)** | **1.37**  **(1.20-1.56)** | **1.44**  **(1.09-1.91)** | **1.84**  **(1.25-2.69)** | **1.56**  **(.125-1.96)** | 1.02  (0.63-1.65) | 0.93  (0.49-1.77) | 0.98  (0.66-1.44) |
| Low income | **1.27**  **(1.07-1.50)** | **1.79**  **(1.43-2.24)** | **1.45**  **(1.27-1.65)** | 1.22  (0.93-1.60) | **1.60**  **(1.11-2.32)** | **1.32**  **(1.07-1.64)** | 1.23  (0.75-2.00) | 1.14  (0.60-2.15) | 1.18  (0.80-1.73) |
| WIHS vs. MACS | **1.31**  **(1.10-1.56)** | **1.66**  **(1.34-2.06)** |  | 0.96  (0.73-1.28) | 1.19  (0.82-1.72) |  | **0.57**  **(0.35-0.91)** | 0.81  (0.42-1.55) |  |

*Note*. PWH indicate PWH. PWoH indicate PWoH. WIHS indicates Women's Interagency HIV Study. MACS indicates the Multicenter AIDS Cohort Study. Low income indicates <$20,000/year for MACS and ≤$18,000/year for WIHS. Northeast (Brooklyn NY, Bronx NY), Mid-Atlantic (Washington DC, Baltimore MD), Midwest (Chicago IL, Pittsburgh PA, Columbus OH), West (San Francisco CA), South (Chapel Hill NC, Atlanta GA, Miami FL, Birmingham AL, Jackson MS).

* Experiencing interruptions 'somewhat' or 'a lot' in mental healthcare.

** Experiencing interruptions 'somewhat' or 'a lot' in substance use treatment.
